# The CCR2^+^ Monocyte Subsets Increase in Obese Boys but Not Girls with Abnormally High Carotid Intima-Media Thickness: A Pilot Study

**DOI:** 10.3390/jcdd9100330

**Published:** 2022-09-29

**Authors:** María José Garcés-Hernández, Karen Pedraza-Escudero, Nayely Garibay-Nieto, Joselin Hernández-Ruiz, Jessica Lakshmi Prieto-Chávez, Lourdes Andrea Arriaga-Pizano, Eréndira Villanueva-Ortega, Galileo Escobedo, Aaron Noe Manjarrez-Reyna, Juan Carlos López-Alvarenga, José Luis Pérez-Hernández, Gloria Queipo-García

**Affiliations:** 1 Childhood Obesity Clinic, Hospital General de México Dr. Eduardo Liceaga, Ciudad de México 06720, Mexico; 2 Clinical Pharmacology Unit, Hospital General de México Dr. Eduardo Liceaga, Dr. Balmis 148, Doctores, Cuauhtémoc, Ciudad de México 06720, Mexico; 3 Flow Cytometry Laboratory, Instrumental Center, Health Investigation Coordination, Hospital de Especialidades del Centro Médico Siglo XXI, Instituto Mexicano del Seguro Social, Ciudad de México 06720, Mexico; 4 Laboratory of Immunometabolism, Research Division, General Hospital of México “Dr. Eduardo Liceaga”, Ciudad de México 06720, Mexico; 5 Population Health and Biostatistics, School of Medicine, University of Texas Rio Grande Valley, Edinburg, TX 78539, USA; 6 Research Department, Universidad México-Americana del Norte, Reynosa 88640, Mexico; 7 Gastroenterology and Hepatology Department, Hospital General de México “Dr. Eduardo Liceaga”, Ciudad de México 06720, Mexico; 8 Department of Human Genetics, Hospital General de México “Dr. Eduardo Liceaga”, Ciudad de México 06720, Mexico

**Keywords:** CCR2, nonclassical monocytes, children, intima-media thickness, insulin resistance, obesity

## Abstract

The differential contribution of monocyte subsets expressing the C-C chemokine receptor 2 (CCR2) to subclinical atherosclerosis in girls and boys is unclear. In this pilot study, we compared classical, intermediate, and nonclassical monocyte subsets expressing CCR2 in 33 obese children of both sexes aged 8 to 16 divided by carotid intima-media thickness (IMT), considering values above the 75th percentile (p75) as abnormally high IMT. Obesity was defined as body mass index above the 95th percentile according to age and sex. Flow cytometry analyses revealed that boys but not girls with IMT ≥ p75 displayed increased CCR2^+^ cell percentage and CCR2 expression in the three monocyte subsets, compared to boys with IMT < p75. The CCR2^+^ cell percentage and CCR2 expression in the three monocyte subsets significantly correlated with increased IMT and insulin resistance in boys but not girls, where the CCR2^+^ nonclassical monocyte percentage had the strongest associations (*r* = 0.73 and *r* = 0.72, respectively). The role of CCR2^+^ monocyte subpopulations in identifying an abnormally high IMT shows a marked sexual dimorphism, where boys seem to be at higher subclinical atherosclerosis risk than girls.

## 1. Introduction

Obesity is a risk factor for coronary heart disease. Cardiovascular diseases cause nearly 70% of deaths among persons with a high body mass index (BMI) [1]. Coronary artery disease, also known as atherosclerosis, is the most common heart disease characterized by the accumulation of cholesterol esters-ingesting macrophages in the subendothelial space of coronary arteries [2].

During obesity, cholesterol levels considerably increase and adhere to the vascular endothelium, triggering the local release of reactive oxygen species and the recruitment of macrophages, which can expand vascular injury and promote atherogenesis [3,4]. Therefore, the atherosclerotic process originates with endothelial damage and dysfunction that lead to the recruitment of circulating monocytes with the ability to adhere to and infiltrate the injured arterial wall [5]. There, monocytes differentiate into macrophages that actively uptake cholesterol esters by phagocytosis, resulting in foam cell formation and further vessel wall inflammation. Foam cells contribute to intima-media thickening (IMT), a condition correlating with increased insulin resistance in children with obesity who can become individuals with advanced atherosclerosis in adult life [6,7,8,9]. In fact, insulin resistance that reflects the progressive loss of the action of insulin on target tissues is also an independent risk factor for developing systemic inflammation that often accompanies atherosclerotic cardiovascular disease [10].

Monocytes are divided into three subpopulations according to CD14 and CD16 expression as follows: classical monocytes (CD14^++^CD16^−^), intermediate monocytes (CD14^++^CD16^+^), and nonclassical monocytes (CD14^+^CD16^+^). The infiltration of monocyte subsets into artery vessels occurs in response to the C-C chemokine receptor 2 (CCR2), a G protein-coupled receptor whose ligands include the monocyte chemoattractant protein (MCP) family [11].

A recent study reported an increase in intermediate monocytes expressing CCR2 in adults with atherosclerosis [11]. CCR2 expression also elicits the adhesion of classical and nonclassical monocytes to the vascular endothelium during atherosclerosis [12]. Another study showed that these monocyte subsets could even help predict an impaired endothelial dysfunction in patients with atherosclerosis in a sex- and age-dependent fashion [13].

Currently, there is evidence of the influence of sex in the risk of developing atherosclerosis. Premenopausal women display less pro-atherogenic lipid profiles than men [14]. Ischemic cardiovascular disease resulting from atherosclerosis occurs more often in men than women. At an early age, male children and adolescents show higher IMT than girls [15]. Sexual dimorphism also influences the CCR2–MCP-1 axis. Petty and colleagues found that African American male adolescents present higher MCP-1 serum levels than females [16], confirming further evidence, indicating that MCP-1 is elevated only in male mice fed a high-fat diet (HFD) [17]. Verweij and coworkers expanded this body of evidence by showing that being a man significantly contributes to an elevated CCR2 expression in circulating monocytes, mostly classical monocytes [18].

Although increasing evidence in adults indicates that monocyte subsets expressing CCR2 contribute to atherosclerosis in a sex-dependent fashion, there is still scant information on girls and boys with obesity. Herein, we conducted a pilot study to analyze CCR2^+^ monocyte subpopulations in children of both sexes with obesity, examining the association of these immune cells with cardiovascular risk factors, such as IMT and insulin resistance in a sex-dependent fashion.

## 2. Materials and Methods

### 2.1. Participants

Initially, 40 children fulfilled the inclusion criteria. After the first biochemical analysis, we identified three volunteers with abnormally high blood cholesterol levels and removed them from the study. Another four volunteers did not agree to provide 6 mL of venous blood, and we withdrew them from the study. In the end, we enrolled 33 children with obesity aged 8 to 16 years in this pilot study. We included all study participants from the Childhood Obesity Clinic at the General Hospital of Mexico between 2017 and 2019. All volunteers agreed to participate in the study by signing the informed consent and assent letters. We excluded from the study participants with a diagnosis of familial hypercholesterolemia, hypertriglyceridemia, or diabetes mellitus, as well as children consuming alcohol or any drug interfering with metabolism and immune response to avoid potential confounding effects on parameters of subclinical atherosclerosis and CCR2 expression in monocyte subpopulations.

We conducted this study after getting approval from the Research, Ethics, and Biosafety Committees for Human Research of the General Hospital of Mexico (registration number: DI/17/311/03/028), following the ethical guidelines of the 1975 Declaration of Helsinki and its posterior amendment in 2013. According to the clinical practice guidelines of the Pediatric Endocrine Society, we used BMI ≥ 95th percentile according to age and sex to diagnose obesity in the studied children [19,20]. We calculated BMI by dividing weight in kilograms by the square of the height in meters.

### 2.2. Anthropometric Measurements

A group of two trained pediatricians and a nutritionist performed all anthropometric measurements in the study population. We measured body weight and composition using the Body Composition Analyzer Model IOI 353 scale (to 0.1 kg). Moreover, we used a stadiometer board mounted on the wall to register height (to the nearest 0.1 cm). We measured waist circumference (WC) using tape in a horizontal line at the midpoint between the lowest rib and the iliac crest. We registered blood pressure with a digital sphygmomanometer, following a fifteen-minute rest. We assessed pubertal development according to the Tanner stages.

### 2.3. Biochemical Markers

We obtained blood samples after 12 h fasting to measure metabolic markers, including glucose, insulin, uric acid, triglycerides, and liver function tests. We measured glucose, aminotransferases, total cholesterol, high density lipoprotein (HDL) cholesterol, low density lipoprotein (LDL) cholesterol, and triglycerides by enzymatic assays, using the Beckman Coulter DxC 700 AU Chemistry Analyzer (Beckman Coulter Inc., Brea, CA, USA). We determined serum insulin by ELISA (Abnova, Corporation, Taipei City, Taiwan).

We calculated the Homeostasis Model Assessment of insulin resistance (HOMA) by multiplying fasting glucose (mg/dL) by fasting insulin (mU/L) divided by 405. We considered children with insulin resistance as having HOMA values ≥ 3.4 [21,22].

### 2.4. Ultrasound Measurements

We used the Siemens Accuson 2000 ultrasound with high resolution (2 and 12 MHz) to get all ultrasound images. We measured abdominal fat by calculating the preperitoneal fat area (PFA). We placed a linear probe perpendicular to the skin of the median upper abdomen, moving the probe longitudinally from the xiphoid process to the umbilicus along with the midline to obtain an image that contained the maximum preperitoneal fat thickness. We considered the PFA as the full height of the triangular-shaped area in the transversal image. We measured the total preperitoneal area starting from the position of the maximum preperitoneal thickness over a distance of 20 mm in the caudal direction [23]. We evaluated the vascular structure as the intima-media thickness (IMT). We measured IMT in a supine position, with the neck extended and the probe in the anterolateral place in a longitudinal plane on the far wall of the left internal carotid artery 1 cm from the carotid bulb. A senior expert radiologist performed five measurements on each study volunteer, using the average IMT value for further analyses. We evaluated the inter-observer variability by calculating Cohen’s kappa index, finding a κ = 0.89, which suggests a high reproducibility when measuring IMT. Depending on age and sex, we defined a high-risk IMT value when it fell above the 75th percentile (p75), based on Böhm et al. [15].

### 2.5. Total Leukocyte Isolation and Flow Cytometry

**Figure 1:**
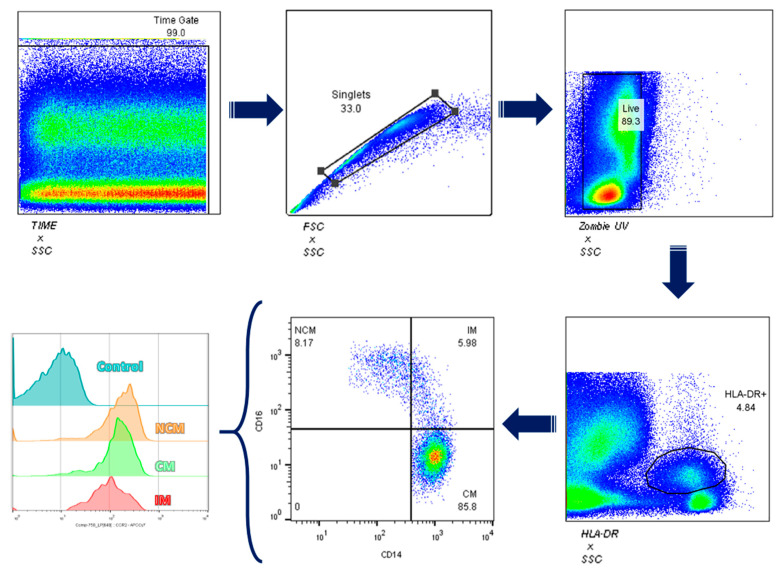
Gating Strategy. We first gated total leukocytes on a time/side scatter density plot and selected the Zombie UV negative cell population for detecting living cells. Next, we gated living cells for singlets on a forward scatter/trigger pulse width density plot. After recognizing cells by size and granularity, we selected monocytes on the HLA-DR gate. Then, we gated monocytes using the rectangular gating strategy on the CD14^+^/CD16^+^ cell population to identify CD14^++^CD16^−^ cells as classical monocytes, CD14^++^CD16^+^ cells as intermediate monocytes, and CD14^+^CD16^+^ cells as nonclassical monocytes (Figure 1). We obtained the median fluorescence intensity (MFI) for CCR2 by considering both positive and negative cell populations. We got the CCR2^+^ cell percentage using fluorescence minus one (FMO) control. For each fluorochrome, we used compensation controls through UltraComp eBeads^TM^ (Invitrogen^TM^, Carlsbad, CA, USA). We analyzed data by the FlowJo 10.0.7 software (TreeStar, Inc., Ashland, OR, USA).

We obtained 6 mL of venous blood from all study participants and placed them in vacutainer tubes treated with EDTA. Immediately after, we centrifuged blood samples at 1200 rpm for 10 min and took the layer of white blood cells to transfer it to 2.5 mL Eppendorf tubes. We removed red blood cells using 1 mL ACK erythrocyte lysis buffer for 7 min at 4 °C. After centrifuging at 2000 rpm/4 °C for 5 min, we discarded the supernatant and obtained the leukocyte fraction. Later, we rinsed the cells with phosphate buffer saline 1X (PBS 1X), and after a centrifugation step at 2000 rpm/4 °C for 5 min, we transferred the cell pellet to 0.6 mL Eppendorf tubes. We counted cells using a Neubauer chamber by the Trypan blue exclusion test to determine the number of viable cells [20]. Next, we transferred leukocytes to an additional 0.6 mL Eppendorf tube at a density of 2 × 10^6^ cells to incubate them with anti-CD14-PECy7 (BD Biosciences, Franklin Lakes, NJ, USA), anti-CD16-PECy5 (BD Biosciences, Franklin Lakes, NJ, USA), and anti-CCR2-Alexa Fluor 647 (BioLegend, San Diego, CA, USA) for 20 min at 4 °C in the absence of light. Then, we rinsed leukocytes with PBS1X and centrifuged samples at 2000 rpm/4 °C for 5 min. After that, we discarded the supernatant and suspended the cell pallet in 200 µL PBS. We acquired cells using the BD FACS Canto II, acquiring 10,000 monocyte events in triplicate. We categorized monocyte subsets according to CD14 and CD16 expression: CD14^++^CD16^−^, classical monocytes; CD14^++^CD16^+^, intermediate monocytes; CD14^+^CD16^+^, nonclassical monocytes (Figure 1). Whereas the percentage of positive cells reflects the number of cells expressing the target molecule, the mean fluorescence intensity (MFI) indicates how much the target molecule is produced in a specific cell population. For this reason, we obtained the MFI for CCR2, considering positive and negative cell populations for this marker. We obtained the percentage of positive cells for CCR2 using proper fluorescence minus one (FMO) controls, using UltraComp eBeads™ (Invitrogen™, Carlsbad, CA, USA) as compensation controls. We performed flow cytometry assays in the same controlled-temperature cell culture room between 9 and 11 in the morning to avoid procedural variations. We analyzed monocyte subpopulations using the FlowJo software version 10.0.

### 2.6. Statistics

We show data as mean ± standard deviation (SD). According to IMT stratification, nine obese girls showed an IMT value above the p75, whereas IMT fell above the p75 in seven obese boys. We used the unpaired student *T*-test to compare demographic, cardiometabolic, and immune variables between girls and boys with and without increased IMT. We used the Pearson correlation test to calculate correlation coefficients with 95% confidence intervals between monocyte subsets and cardiometabolic outcomes. We used the rank-based inverse-normal transformation to estimate the interaction levels in the regression for sex, considering age and other metabolic and cellular variables. We adjusted data by sex, age, and sex–age interaction in multiple regression analysis, considering a minimum of ten subjects per variable to estimate regression coefficients, standard errors, and confidence intervals. Kappa calculation for IMT measurements was used to evaluate reproducibility of the data. We considered a *p* value < 0.05 as significant. We analyzed data using SPSS version 22.0 and the GraphPad Prism 6.01 software (GraphPad Software, La Jolla, CA 92037, USA).

## 3. Results

### 3.1. Participants Characteristics

We enrolled 33 children with obesity with an average age of 10.9 ± 2.1, where 57.5% were females. According to sex, we found differences in body composition, where females showed a significant 4.6% increase in body fat percentage (BFP), compared to males (*p* = 0.008). Conversely, males presented 33.4% more visceral fat adiposity (VFA), compared to females (*p* = 0.002) (Table 1). There were no differences between females and males for BMI, WC, blood pressure, and PFA (Table 1). Children of both sexes presented similar pubertal stages and glucose homeostasis, showing similar blood glucose values, glycosylated hemoglobin, insulin, and HOMA (Table 1). Furthermore, there were no differences between females and males for uric acid, lipid profile, and liver function tests (Table 1). Children of both sexes also displayed similar IMT values, even after adjusting data by sex, age, or the interaction between these two variables (adjusted R square = 0.252).

### 3.2. CCR2^+^ Monocyte Subsets in Boys and Girls with Obesity and High Risk IMT

**Figure 2:**
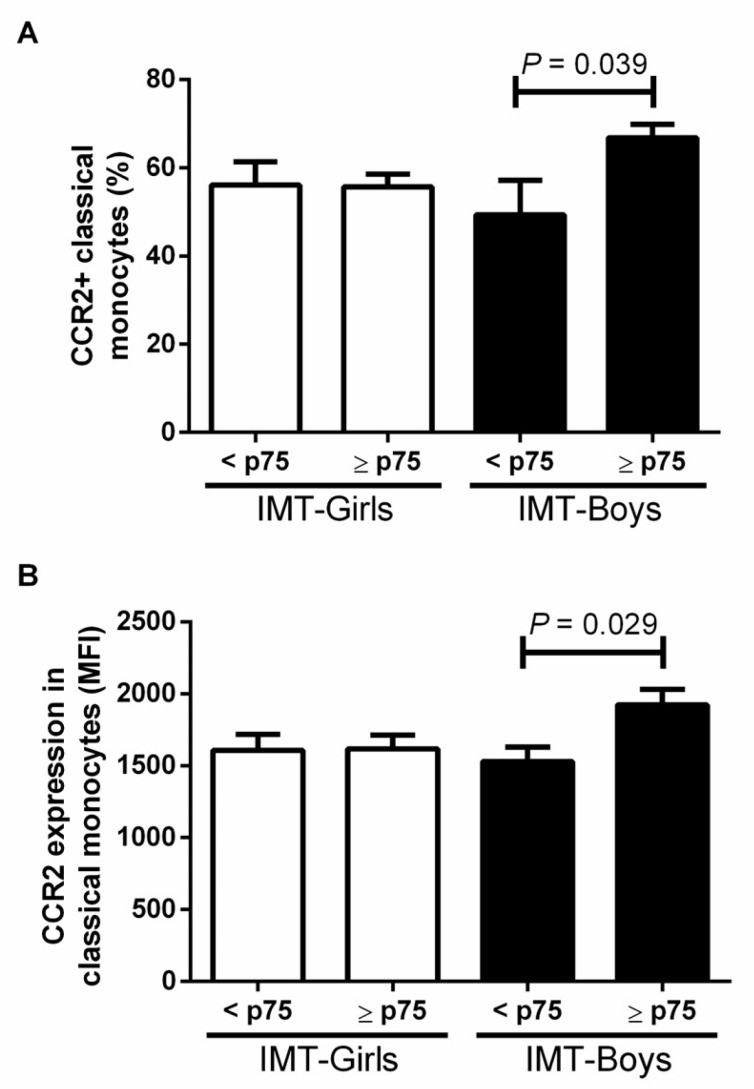
Evaluation of (**A**) the proportion of CCR2 in classical monocytes and intima media thickness (IMT) and (**B**) expression of CCR2 in classical monocytes and intima media thickness (IMT) by sex. We considered a high-risk IMT ≥ p75 and compared differences by the student *T*-test, considering a *p* value < 0.05 as significant.

Among all study participants, seven boys and nine girls showed IMT values above the p75. We found an overall increase in both CCR2^+^ monocyte percentage (37.3 ± 17.4%, *p* = 0.017) and CCR2 expression (20.2 ± 4.3%, *p* = 0.017) in male obese children, compared to females. The CCR2^+^ classical monocyte percentage was 14.7 ± 9.47% higher in male obese children with subclinical atherosclerosis risk than in boys with IMT < p75 (*p* = 0.039). Moreover, CCR2 expression in this monocyte subset was 20.3% ± 1.02% higher in male obese children with IMT ≥ p75 than in boys with IMT values below the p75 (*p* = 0.029) (Figure 2).

**Figure 3:**
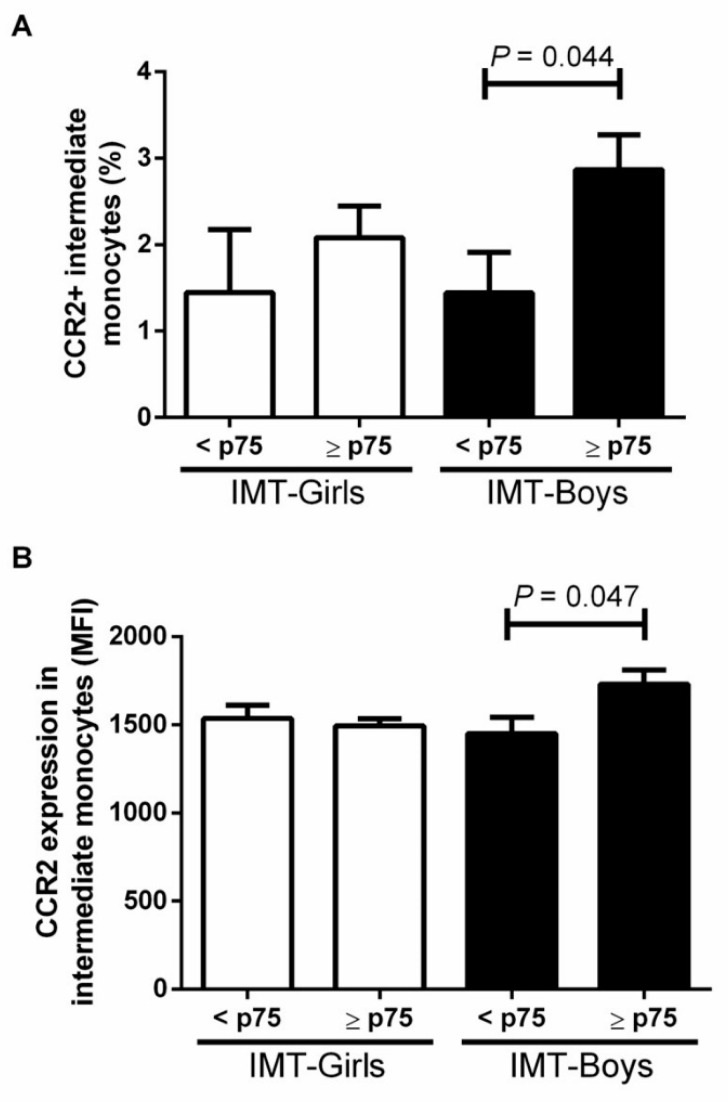
Evaluation of (**A**) the proportion of CCR2 in intermediate monocytes and intima media thickness (IMT) and (**B**) expression of CCR2 in intermediate monocytes and intima media thickness (IMT) by sex. We considered a high-risk IMT ≥ p75 and compared differences by the student *T*-test, considering a *p* value < 0.05 as significant.

The intermediate monocyte subpopulation displayed similar behavior, where the CCR2^+^ cell percentage was 50 ± 36.7% higher in male children with IMT ≥ p75 than in boys with IMT below the p75 (*p* = 0.044). The CCR2 expression was 16.1% ± 6.5% higher in intermediate monocytes from boys with IMT ≥ p75 than in male children without subclinical atherosclerosis risk (*p* = 0.047) (Figure 3).

**Figure 4:**
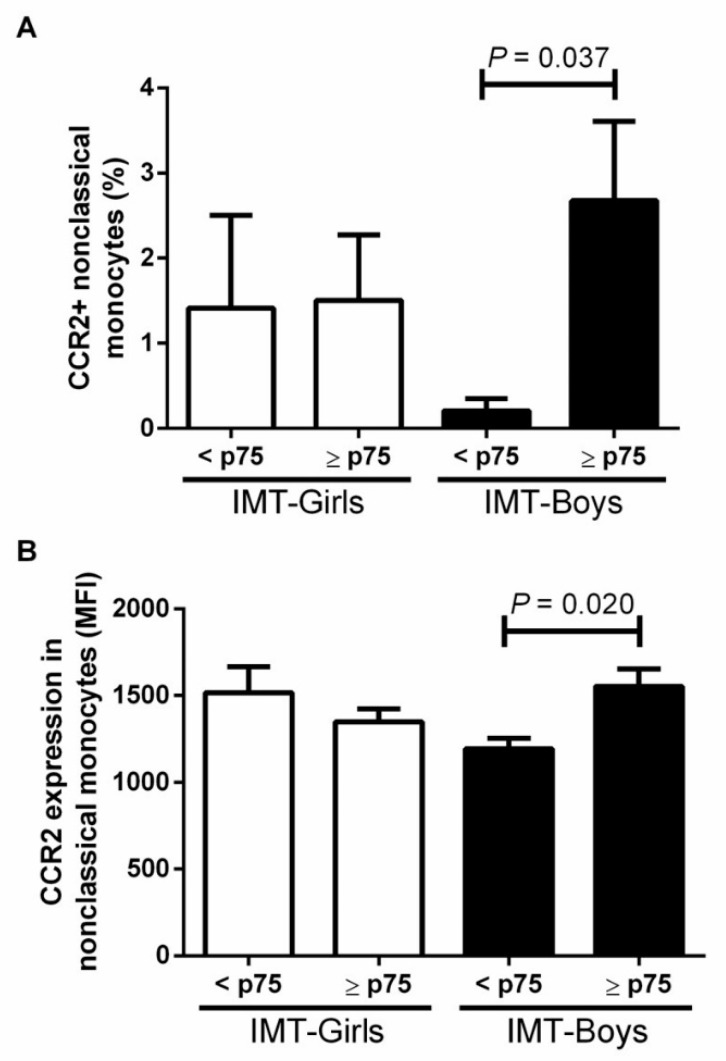
Evaluation of (**A**) the proportion of CCR2 in nonclassical monocytes and intima media thickness (IMT) and (**B**) expression of CCR2 in nonclassical monocytes and intima media thickness (IMT) by sex. We considered a high-risk IMT ≥ p75 and compared differences by the student *T*-test, considering a *p* value < 0.05 as significant.

The proportion of nonclassical monocyte expressing CCR2 was 92.3± 80.1% higher in male children with IMT ≥ p75 than in those without a high-risk IMT (*p* = 0.037) (Figure 4). Moreover, the CCR2 MFI was 23.1 ± 8.4% higher in nonclassical monocytes from male children with high cardiovascular risk than in males with normal IMT (*p* = 0.020) (Figure 4). We found no differences in obese girls with IMT below the p75.

### 3.3. Correlation between CCR2^+^ Monocyte Subsets and Children with Obesity and High Risk IMT

Furthermore, we explored potential associations of classical, intermediate, and nonclassical monocytes expressing CCR2 with cardiovascular and metabolic risk factors, such as HOMA and IMT. Interestingly, we noticed that males but not females displayed a strong positive correlation of CCR2^+^ circulating monocytes with IMT ≥ p75 (Table 2). We also found that CCR2^+^ classical, intermediate, and nonclassical monocyte percentage and CCR2 expression in these three monocyte subsets correlated with higher IMT values in males but not females (Table 2). The CCR2^+^ nonclassical monocyte percentage had the strongest association with IMT in males but not females (*r* = 0.72, *p* < 0.01). Additionally, we observed a strong correlation of CCR2^+^ nonclassical monocytes with insulin resistance (*r* = 0.80, *p* < 0.01) in males with IMT ≥ p75 but not females with the same criterion for subclinical atherosclerosis (Table 2). We did not observe significant correlations between monocyte subsets expressing CCR2^+^ with IMT or insulin resistance levels in female children, even those with IMT ≥ p75 (Table 2).

## 4. Discussion

The contribution of sexual dimorphism in synergy with monocyte subpopulations expressing CCR2 to the development of atherosclerosis, since early ages is still unclear. Studies in animal models and human adults show that CCR2 expression in monocytes is associated with increased arterial wall inflammation and atherosclerosis progression, where males exhibit more robust CCR2 expression than females [15]. Herein, we used IMT ≥ p75 adjusted by sex and age as an early surrogate marker of subclinical atherosclerosis and cardiovascular risk in children with obesity instead of using a cutoff value of IMT that could lead us to bias and data misinterpretation [15]. In this scenario, we found that only boys with IMT ≥ p75 showed increased CCR2 expression in classical, intermediate, and nonclassical monocytes, compared to girls that also exhibited considerably high IMT values. In line with our results, two independent studies previously reported that compared to females, male adolescents display higher circulating levels of MCP-1, a CCR2 ligand whose interaction stimulates monocyte mobilization toward damaged tissues [16,24]. Simoes and coworkers observed that boys with obesity showed increased serum amyloid A (SAA) values, compared to girls; SAA induces MCP-1 production in monocytes and is associated with a higher risk of atherosclerosis [25,26]. These findings suggest that the atherosclerotic process may begin early in childhood in a sex-dependent fashion, resulting from the interaction between obesity, monocyte activation, and intima-media progressive thickening. As mentioned, the binding of the chemokine MCP-1 to CCR2 in circulating monocytes is one of the earliest steps in atherogenesis and is intimately linked to IMT continuing enlargement [27,28]. Okumoto and coworkers reported that monocytes expressing CCR2 correlate with increased IMT and are an independent risk factor promoting atherogenesis [29]. In this study, we observed a strong positive correlation of CCR2 expression in all monocyte subsets with IMT in male children but not in girls. Notably, nonclassical monocytes from boys with elevated IMT exhibited the highest increment in CCR2 expression, compared to male children with average IMT values and girls with and without IMT ≥ p75. Nonclassical and classical monocytes are recruited to vascular fatty streaks to form the atherosclerotic plaque, where classical monocytes migrate into the vascular wall, and nonclassical monocyte subsets remain in the lumen exerting patrolling functions [30]. It is feasible that the increase in nonclassical monocytes expressing CCR2 may reflect an attempt of these immune cells to repair the injured vascular endothelium, a notion suggesting the use of these cells as an early marker of subclinical atherosclerosis in obese boys.

The sexual dimorphism we saw in this work is supported by other studies in adults where monocyte subsets are linked to increased IMT in a sex-dependent fashion. Cannon and coworkers observed a positive correlation between IMT and nonclassical monocytes, where men showed a higher nonclassical monocyte percentage than women [31]. Feinstein and coworkers recently reported in male adults but not females that nonclassical monocytes correlate with IMT expansion during a ten-year follow-up [32]. Rogacev and coworkers found a higher classical monocyte percentage in male patients subjected to coronary angiography than in women facing the same procedure [33]. Altogether, this information expands the knowledge behind the increased risk of developing atherosclerosis in males with obesity, compared to overweighed females, where monocyte subsets dynamically contribute to plaque formation. Studies in adult subjects found that classical and intermediate monocytes could predict adverse cardiovascular events [33,34]. Feinstein and coworkers reported that nonclassical monocytes could be an early biomarker of pre-atherosclerotic vascular lesion formation [32]. Larger studies in children are needed to evaluate the use of circulating monocyte subpopulations as accurate predictors of subclinical atherosclerosis.

Insulin resistance is defined as a reduce response of insulin-target tissues, such as the liver, skeletal muscle, and visceral fat to insulin action [35]. Insulin resistance leads to persistent hyperglycemia and is a risk factor intimately linked to atherosclerosis development. In an insulin-resistant setting, a reduced nitrite oxide (NO) bioavailability induces vascular damage, increasing the expression of adhesion molecules and inflammatory cytokines with the ability to promote monocyte recruitment and infiltration to the blood vessel wall [36]. It was our rationale behind estimating insulin resistance in our cohort of obese children, analyzing potential associations of monocyte subsets, expressing CCR2 with IMT and insulin resistance. Other studies in adult individuals looked for similar correlations between circulating monocytes and markers of altered glucose homeostasis associated with atherosclerosis progression. Poitou and coworkers observed that nonclassical monocytes were independently associated with fasting glycemia [37]. Mine and coworkers found increased CCR2 expression in circulating monocytes from diabetic patients, compared to control subjects, informing a direct correlation of CCL2 serum levels with glycated hemoglobin (HgA1c) [38]. Gallego-Suárez and coworkers reported a positive correlation of classical monocytes with HOMA in children with high BMI [39]. In line with these findings, we found a strong positive correlation of CCR2 expression in nonclassical monocytes with insulin resistance only in male children with IMT > p75. We encourage other research teams to assess whether the interaction between monocyte subsets expressing CCR2 and insulin resistance, as surrogate markers of subclinical atherosclerosis, can help understand the development of subclinical atherosclerosis early in life. Recently, research in this field is gaining attention because of novel therapeutic approach that blocks the CCL2–CCR2 axis and may improve atherosclerosis disease [40].

Due to the increasing obesity rates, cardiovascular events are still the leading cause of mortality worldwide, above all in men. Ischemic cardiovascular disease caused by atherosclerosis is more prevalent and often occurs earlier in men than in women [14]. Cardiovascular diseases are linked to insulin resistance and T2D, which are more prevalent in males than females [41]. Our findings in obese boys with IMT above average values indicate that sexual dimorphism can appear early in life. Sex differences in body composition exist even before exposure to gonadal hormones [42]. Males cumulate more visceral adipose tissue (VAT), whereas females amass more subcutaneous adipose tissue (SAT) [43]. Numerous lines of research have consistently reported that VAT gain triggers low-grade inflammation and monocyte activation, increasing the risk of developing insulin resistance, T2D, atherosclerosis, and ischemic heart disease [44]. In line with these findings, we observed that male children with obesity had more VAT than females, which could partially explain the increase in CCR2 expression in circulating monocytes from boys with IMT > p75.

This pilot study has some limitations. First, although statistically acceptable, the sample size appears small to estimate whether potentially confounding variables, such as sex or sexual development, may impact the measurement of intima-media thickness. To lessen the effect of this limitation, we adjusted data by age, sex, and the interaction of sex with age in multiple regression models, finding that only age but not sex appears to influence IMT. Another limitation of the study is that we analyzed body composition using a Body Composition Analyzer Model IOI 353 scale suitable for examining the pediatric population. However, if available, we would have liked to evaluate body composition using dual-energy X-ray absorptiometry (DEXA) or magnetic resonance imaging (MRI). Conversely, a strength of the work is that children enrolled in the study had no previous pharmacological treatment that could influence CCR2 expression, monocyte subset dynamic, or IMT values. We are now working on increasing the number of children enrolled in the study to clarify the contribution of circulating monocytes expressing CCR2 and other chemokine receptor repertoires to atherosclerosis development in a sex-dependent fashion.

## 5. Conclusions

In conclusion, CCR2 expression in circulating monocyte subsets is associated with elevated IMT and insulin resistance in male children with obesity. Circulating monocytes expressing CCR2, mostly nonclassical monocytes, may contribute to atherosclerotic plaque formation in a sex-dependent fashion, where boys appear to be at increased cardiovascular risk.

## Figures and Tables

**Table 1:** Demographic, anthropometric, biochemical, and cardiovascular characteristics of the study participants.

	All Participants (*n* = 33)	Girls ^a^ (*n* = 19)	Boys ^b^ (*n* = 14)	*p* ^a vs. b^
Anthropometric variables				
Age (years)	10.9 ± 2.1	11.0 ± 2.4	10.7 ± 1.7	0.741
Weight (kg)	59.1 ± 15.1	57.8 ± 14.9	60.9 ± 15.8	0.559
Height (cm)	147.6 ± 10.8	145.5 ± 11.32	150.4 ± 9.8	0.210
BMI (kg/m^2^)	26.6 ± 3.3	26.8 ± 2.9	26.4 ± 3.8	0.786
Waist (cm)	86.5 ± 10.3	84.2 ± 8.3	89.7 ± 12.2	0.134
SBP(mmHg)	103.3 ± 9.8	101.5 ± 9.7	105.6 ± 9.8	0.247
DBP (mmHg)	68.7 ± 8.7	66.6 ± 7.0	71.5 ± 10.1	0.118
BFP (%)	31.4 ± 5.0	33.3 ± 3.8	28.7 ± 5.4	**0.008**
VFA (cm^3^)	86.5 ± 34.5	71.3 ± 22.9	107.1 ± 37.5	**0.002**
PFA (cm^2^)	4.6 ± 1.6	4.5 ± 1.9	4.6 ± 1.1	0.860
Puberty Stages				
Tanner Stage	43.3%	33.3%	58.3%	0.111
1				
2	20%	16.7%	25%	
3	10%	5.6%	26.7%	
4	23.3%	38.9%	0%	
5	3.3%	5.6%	0%	
Glucose Homeostasis				
Glucose (mg/dL)	88.6 ± 8.4	88.9 ± 8.7	86.8 ± 8.2	0.493
HbA1c	5.3 ± 0.2	5.2 ± 0.1	5.3 ± 0.2	0.183
Insulin (mg/dL)	16.4 ± 1.4	16.9 ± 7.2	15.6 ± 10.2	0.687
HOMA	3.7 ± 1.9	4.0 ± 1.7	3.4 ± 2.3	0.403
Biochemical variables				
Uric acid (mg/dL)	5.7 ± 1.0	5.8 ± 1.0	5.6 ± 1.1	0.958
TG (mg/dL)	138.5 ± 54.5	133.4 ± 45.3	145.4 ± 66.3	0.567
ALT (UI/L)	24.8 ± 1.8	22.8 ± 8.5	27.5 ± 13.3	0.235
AST (UI/L)	28.1 ± 7.2	26.3 ± 6.8	30.5 ± 6.8	0.105
GGT (UI/L)	16.2 ± 0.9	14.9 ± 3.7	18.0 ± 6.5	0.134
Cardiovascular variables				
Tot Chol (mg/dL)	163.7 ± 21.0	162.0 ± 18.6	166.0 ± 24.5	0.603
HDL (mg/dL)	40.0 ± 7.8	40.0 ± 8.0	39.9 ± 7.8	0.965
LDL (mg/dL)	107.2 ± 17.9	107.0 ± 16.5	107.5 ± 20.3	0.938
LDL/HDL Ratio	2.7 ± 0.1	2.7 ± 0.6	2.7 ± 0.7	0.924
IMT (mm)	0.56 ± 0.1	0.58 ± 0.10	0.55 ± 0.11	0.399

Values are expressed as mean (standard deviation) for normal distribution. *T*-test analysis, Bold data are *p* values < 0.05. BMI: body mass index, SDB: systolic blood pressure, DPB: diastolic blood pressure, Tot Chol: total cholesterol, HbA1c: glycated hemoglobin, HOMA: homeostasis model assessment, HDL: high density lipoprotein cholesterol, LDL: low density lipoprotein cholesterol, TG: triglycerides, ALT: alanine aminotransferase, AST: aspartate aminotransferase, GGT: gamma glutamyl transferase, BFP: body fat percentage, VFA: visceral fat adiposity, PFA: preperitoneal fat area, IMT: intima media thickness. “^a^” and “^b^” are placed to represent girls and boys and identify that the *p* value is the result of the comparison of both.

**Table 2:** Correlation of monocyte subsets expressing CCR2 with IMT and HOMA in boys and girls with obesity.

		CCR2 %		CCR2 MFI	
		IMT ≥ p75	HOMA ≥ 3.4	IMT ≥ p75	HOMA ≥ 3.4
CM	Boys (*n* = 14)	*r* = 0.56 * **(0.07–0.85)**	*r* = 0.21 (−0.45–0.72)	*r* = 0.61 * **(0.20–0.86)**	*r* = 0.22 (−0.45–0.70)
	Girls (*n* = 19)	*r* = −0.05 (−0.68–0.58)	*r* = −0.11 (−0.72–0.60)	*r* = 0.22 (−0.36–0.72)	*r* = −0.21 (−0.88–0.42)
IM	Boys (*n* = 14)	*r* = 0.62 * **(0.17–0.88)**	*r* = 0.41 (−0.12–0.88)	*r* = 0.62 * **(0.19–0.91)**	*r* = 0.52 (−0.00–0.86)
	Girls (*n* = 19)	*r* = 0.27 (−0.27–0.88)	*r* = 0.03 (−0.62–0.80)	*r* = −0.04 (−0.60–0.48)	*r* = −0.07 (−0.68–0.50)
NCM	Boys (*n* = 14)	*r* = 0.72 ** **(0.38–0.91)**	*r* = 0.80 ** **(0.60–0.93)**	*r* = 0.73 ** **(0.45–0.91)**	*r* = 0.70 ** **(0.40–0.90)**
	Girls (*n* = 19)	*r* = 0.15 (−0.39–0.74)	*r* = −0.22 (−0.71–0.40)	*r* = −0.21 (−0.75–0.33)	*r* = −0.20 (−0.69–0.37)

CCR2 (%) indicates the percentage of monocytes expressing CCR2, while CCR2 (MFI) denotes how big CCR2 is expressed in each monocyte subset. We considered children with IMT ≥ p75 to have a higher risk for subclinical atherosclerosis. We considered HOMA ≥ 3.4 as a higher risk for insulin resistance. The number of girls we analyzed was 19, whereas we incorporated 14 boys into the study. The table shows correlation coefficient values (*r*) that we calculated by Pearson’s correlation coefficient with 95% confidence intervals written between parentheses. We considered significant values when *p* < 0.05, indicating them in bold as follows: * *p* < 0.05, ** *p* < 0.001. CM, classical monocytes; IM, intermediate monocytes; NCM, nonclassical monocytes; MFI, mean fluorescent intensity; IMT, intima-media thickness; HOMA, homeostasis model assessment for insulin resistance.

## Data Availability

Data are available on reasonable request to the corresponding author.

## References

[1] Afshin A., Forouzanfar M.H., Reitsma M.B., Sur P., Estep K., Lee A., Marczak L., Mokdad A.H., Moradi-Lakeh M., GBD 2015 Obesity Collaborators (2017). Health Effects of Overweight and Obesity in 195 Countries over 25 Years. N. Engl. J. Med..

[2] Medina-leyte D.J., Zepeda-garc O., Dom M. (2021). Endothelial Dysfunction, Inflammation and Coronary Artery Disease: Potential Biomarkers and Promising Therapeutical Approaches. Int. J. Mol. Sci..

[3] Hajjar D.P., Gotto A.M. (2013). MINI-REVIEW Biological Relevance of In fl ammation and Oxidative Stress in the Pathogenesis of Arterial Diseases. Am. J. Pathol..

[4] Rocha V.Z., Libby P. (2009). Obesity, inflammation, and atherosclerosis. Nat. Rev. Cardiol..

[5] Poznyak A., Grechko A.V., Poggio P., Myasoedova V.A., Alfieri V., Orekhov A.N. (2020). The diabetes mellitus–atherosclerosis connection: The role of lipid and glucose metabolism and chronic inflammation. Int. J. Mol. Sci..

[6] Powell-Wiley T.M., Poirier P., Burke L.E., Després J.-P., Gordon-Larsen P., Lavie C.J., Lear S.A., Ndumele C.E., Neeland I.J., Sanders P. (2021). Obesity and Cardiovascular Disease: A Scientific Statement From the American Heart Association. Circulation.

[7] McGill H.C., McMahan C.A., Herderick E.E., Zieske A.W., Malcom G.T., Tracy R.E., Strong J.P. (2002). Obesity accelerates the progression of coronary atherosclerosis in young men. Circulation.

[8] Giannini C., Santoro N., Caprio S., Kim G., Lartaud D., Shaw M., Pierpont B., Weiss R. (2011). The triglyceride-to-HDL cholesterol ratio: Association with insulin resistance in obese youths of different ethnic backgrounds. Diabetes Care.

[9] Drozdz D., Alvarez-Pitti J., Wójcik M., Borghi C., Gabbianelli R., Mazur A., Herceg-čavrak V., Lopez-Valcarcel B.G., Brzeziński M., Lurbe E. (2021). Obesity and cardiometabolic risk factors: From childhood to adulthood. Nutrients.

[10] Di Pino A., Defronzo R.A. (2019). Insulin Resistance and Atherosclerosis: Implications for Insulin-Sensitizing Agents. Endocr. Rev..

[11] Moroni F., Ammirati E., Norata G.D., Magnoni M., Camici P.G. (2019). The role of monocytes and macrophages in human atherosclerosis, plaque neoangiogenesis, and atherothrombosis. Med. Infl..

[12] França C.N., Izar M.C.O., Hortêncio M.N.S., do Amaral J.B., Ferreira C.E.S., Tuleta I.D., Fonseca F.A.H. (2017). Monocyte subtypes and the CCR2 chemokine receptor in cardiovascular disease. Clin. Sci..

[13] Urbanski K., Ludew D., Filip G., Filip M., Sagan A., Szczepaniak P., Grudzien G. (2017). CD14+CD16++ “nonclassical” monocytes are associated with Endothelial dysfunction in patients with coronary artery disease. Thromb. Haemost..

[14] Man J.J., Beckman J.A., Jaffe I.Z. (2020). Sex as a Biological Variable in Atherosclerosis. Circ. Res..

[15] Böhm B., Hartmann K., Buck M., Oberhoffer R. (2009). Sex differences of carotid intima-media thickness in healthy children and adolescents. Atherosclerosis.

[16] Petty K.H., Li K., Dong Y., Fortenberry J., Stallmann-Jorgensen I., Guo D., Zhu H. (2010). Sex dimorphisms in inflammatory markers ad adiposity in African American youth. Int. J. Ped. Obes..

[17] Dhanraj P., van Heerden M.B., Pepper M.S., Ambele M.A. (2021). Sexual dimorphism in changes that occur in tissues, organs and plasma during the early stages of obesity development. Biology.

[18] Verweij S.L., Duivenvoorden R., Stiekema L.C.A., Nurmohamed N.S., Van Der Valk F.M., Versloot M., Verberne H.J., Stroes E.S.G., Nahrendorf M., Bekkering S. (2018). CCR2 expression on circulating monocytes is associated with arterial wall inflammation assessed by 18F-FDG PET/CT in patients at risk for cardiovascular disease. Cardiovasc. Res..

[19] Kuczmarski R.J., Ogden C.L., Guo S.S., Grummer-Strawn L.M., Flegal K.M., Mei Z., Wei R., Curtin L.R., Roche A.F., Johnson C.L. (2002). 2000 CDC growth charts for the United States: Methods and development. Vital Health Stat..

[20] Styne D.M., Arslanian S.A., Connor E.L., Farooqi I.S., Murad M.H., Silverstein J.H., Yanovski J.A. (2017). Pediatric Obesity—Assessment, Treatment, and Prevention: An Endocrine Society Clinical Practice Guideline. J. Clin. Endo. Metabo..

[21] Lentferink Y.E., Elst M.A.J., Knibbe C.A.J., Vorst M.M.J. (2017). Van Der. Predictors of Insulin Resistance in Children versus Adolescents with Obesity. J. Obes..

[22] Van Der Aa M.P., Farsani S.F., Kromwijk L.A.J., De Boer A., Knibbe C.A.J., Van Der Vorst M.M.J. (2014). How to Screen Obese Children at Risk for Type 2 Diabetes Mellitus?. Clin. Pediatr..

[23] Mook-Kanamori D.O., Holzhauer S., Hollestein L.M., Durmus B., Manniesing R., Koek M., Boehm G., van der Beek E.M., Hofman A., Witteman J.C.M. (2009). Abdominal Fat in Children Measured by Ultrasound and Computed Tomography. Ultrasound Med. Biol..

[24] Herder C., Schneitler S., Rathmann W., Haastert B., Schneitler H., Winkler H., Bredahl R., Hahnloser E., Martin S. (2007). Low-grade inflammation, obesity, and insulin resistance in adolescents. J. Clin. Endocrinol. Metab..

[25] Lee H.Y., Kim S.D., Shim J.W., Lee S.Y., Lee H., Cho K.-H., Yun J., Bae Y.-S. (2008). Serum Amyloid A Induces CCL2 Production via Formyl Peptide Receptor-Like 1-Mediated Signaling in Human Monocytes. J. Immunol..

[26] Simoes E., Correia-Lima J., Sardas L., Storti F., dos Santos Otani T.Z., Vasques D.A.C., Otani V.H.O., Bertolazzi P., Kochi C., Seelaender M. (2021). Sex dimorphism in inflammatory response to obesity in childhood. Int. J. Obes..

[27] Kang H., Li X., Xiong K., Song Z., Tian J., Wen Y., Sun A., Deng X. (2021). The Entry and Egress of Monocytes in Atherosclerosis: A Biochemical and Biomechanical Driven Process. Cardiovas. Therap..

[28] Tan C., Liu Y., Li W., Deng F., Liu X., Wang X., Gui Y., Qin L., Hu C., Chen L. (2014). Associations of matrix metalloproteinase-9 and monocyte chemoattractant protein-1 concentrations with carotid atherosclerosis, based on measurements of plaque and intima-media thickness. Atherosclerosis.

[29] Okumoto S., Taniguchi Y., Nakashima A., Masaki T., Ito T., Ogawa T., Takasugi N., Kohno N., Yorioka N. (2009). C-C chemokine receptor 2 expression by circulating monocytes influences atherosclerosis in patients on chronic hemodialysis. Therap. Apher. Dial..

[30] Narasimhan P.B., Marcovecchio P., Hamers A.A.J., Hedrick C.C. (2019). Nonclassical Monocytes in Health and Disease. Annu. Rev. Immunol..

[31] Cannon J.G., Sharma G., Sloan G., Dimitropoulou C., Baker R.R., Mazzoli A., Kraj B., Mulloy A., Cortez-Cooper M. (2014). Leptin regulates CD16 expression on human monocytes in a sex-specific manner. Physiol. Rep..

[32] Feinstein M.J., Doyle M.F., Stein J.H., Sitlani C.M., Fohner A.E., Huber S.A., Landay A.L., Heckbert S.R., Rice K., Kronmal R.A. (2021). Nonclassical Monocytes (CD14dimCD16+) Are Associated with Carotid Intima-Media Thickness Progression for Men but Not Women: The Multi-Ethnic Study of Atherosclerosis—Brief Report. Arter. Thromb Vasc Biol..

[33] Rogacev K.S., Cremers B., Zawada A.M., Seiler S., Binder N., Ege P., Große-Dunker G., Heisel I., Hornof F., Jeken J. (2012). CD14++CD16+ monocytes independently predict cardiovascular events: A cohort study of 951 patients referred for elective coronary angiography. J. Am. Coll. Cardiol..

[34] Berg K.E., Ljungcrantz I., Andersson L., Bryngelsson C., Hedblad B., Fredrikson G.N., Nilsson J., Björkbacka H. (2012). Elevated CD14++CD16-monocytes predict cardiovascular events. Circ. Cardiovasc Gen..

[35] Lee S.H., Park S.Y., Choi C.S. (2022). Insulin Resistance: From Mechanisms to Therapeutic Strategies. Diabetes Metab. J..

[36] Di Pietrantonio N., Palmerini C., Pipino C., Baldassarre M.P.A., Bologna G., Mohn A., Giannini C., Lanuti P., Chiarelli F., Pandolfi A. (2021). BBA—Molecular Basis of Disease Plasma from obese children increases monocyte-endothelial adhesion and affects intracellular insulin signaling in cultured endothelial cells: Potential role of mTORC1-S6K1. BBA Mol. Basis Dis..

[37] Poitou C., Dalmas E., Renovato M., Benhamo V., Hajduch F., Abdennour M., Kahn J.-F., Veyrie N., Rizkalla S., Fridman W.-H. (2011). CD14dim CD16+ and CD14+CD16+Monocytes in obesity and During Weight Loss Relationships With Fat Mass and Subclinical Atherosclerosis. Arter. Thromb. Vasc. Biol..

[38] Mine S., Okada Y., Tanikawa T., Kawahara C., Tabata T., Tanaka Y. (2006). Increased expression levels of monocyte CCR2 and monocyte chemoattractant protein-1 in patients with diabetes mellitus. Biochem. Biophys. Res. Commun..

[39] Gállego-Suárez C., Bulan A., Hirschfeld E., Wachowiak P., Abrishami S., Griffin C., Sturza J., Tzau A., Hayes T., Woolford S.J. (2020). Enhanced Myeloid Leukocytes in Obese Children and Adolescents at Risk for Metabolic Impairment. Front. Endocrinol..

[40] Georgakis M.K., Bernhagen J., Heitman L.H., Weber C., Dichgans M. (2022). Targeting the CCL2–CCR2 axis for atheroprotection. Eur. Heart J..

[41] McEwan S., Kwon H., Tahiri A., Shanmugarajah N., Cai W., Ke J., Huang T., Belton A., Singh B., Wang L. (2021). Deconstructing the origins of sexual dimorphism in sensory modulation of pancreatic β cells. Mol. Metab..

[42] Zore T., Palafox M., Reue K. (2018). Sex differences in obesity, lipid metabolism, and inflammation—A role for the sex chromosomes?. Mol. Metab..

[43] Chang E., Varghese M., Singer K. (2018). Gender and Sex Differences in Adipose Tissue. Curr. Diab. Rep..

[44] Neeland I.J., Ross R., Després J.-P., Matsuzawa Y., Yamashita S., Shai I., Seidell J., Magni P., Santos R.D., Arsenault B. (2019). Visceral and ectopic fat, atherosclerosis, and cardiometabolic disease: A position statement. Lancet Diab. Endocrinol..

